# SP13786通过CAFs外泌体抑制Stat3-EMT影响肺腺癌细胞A549的迁移侵袭

**DOI:** 10.3779/j.issn.1009-3419.2021.104.07

**Published:** 2021-06-20

**Authors:** 淑淑 王, 镓钰 崔, 凯佳 张, 金华 谷, 远航 郑, 宝刚 张, 立宏 史

**Affiliations:** 1 261053 潍坊，潍坊医学院山东省应用药理学重点实验室 Shandong Province Key Laboratory of Applied Pharmacology, Weifang Medical University, Weifang 261053, China; 2 261053 潍坊，潍坊医学院临床医学院 College of Clinical Medicine, Weifang Medical University, Weifang 261053, China

**Keywords:** 肺腺癌, SP13786, 肿瘤相关成纤维细胞, 成纤维细胞活化蛋白, 外泌体, EMT, Stat3, Lung Adenocarcinoma, SP13786, Cancer-associated fibroblasts, Fibroblast activation protein, Exosomes, EMT, Stat3

## Abstract

**背景与目的:**

成纤维细胞活化蛋白（fibroblast activation protein, FAP）是肿瘤相关成纤维细胞（cancer-associated fibroblasts, CAFs）的表面标志物之一，与CAFs的恶性表征关系密切，SP13786是FAP的特异性小分子抑制剂。本研究探讨SP13786作用于CAFs后，CAFs外泌体（exosomes, exo）对A549细胞迁移、侵袭的影响与机制。

**方法:**

原代提取CAFs和癌旁成纤维细胞（peri-tumer fibroblasts, PTFs）；MTT实验检测不同浓度SP13786对CAFs增殖的影响；聚合物沉淀法提取PTFs-exo、CAFs-exo以及CAFs+SP13786-exo。将A549细胞设对照组、PTFs组、CAFs组及CAFs+SP13786组并分别以等体积的DMEM、PTFs-exo、CAFs-exo及CAFs+SP13786-exo孵育细胞。激光共聚焦实验检测A549细胞摄取外泌体的情况；免疫荧光、免疫组化和Western blot方法检测α平滑肌肌动蛋白（alpha-smooth muscle actin, α-SMA）、FAP在PTFs和CAFs中的表达及E-cadherin、N-cadherin、Slug、Stat3、P-Stat3在各组A549细胞中的表达；划痕实验和Transwell实验检测各组细胞的迁移和侵袭能力。

**结果:**

免疫荧光、免疫组化和Western blot结果均显示α-SMA、FAP在CAFs中高表达，在PTFs中低表达（*P* < 0.05），表明从肺腺癌组织和癌旁组织中分别成功获得了CAFs和PTFs。MTT实验测得SP13786对于CAFs细胞的半数抑制浓度（50% inhibitory concentration, IC_50_）约为3.3 nmol/L。免疫组化和Western blot结果显示与CAFs组相比，CAFs+SP13786组的α-SMA与FAP的表达显著降低（*P* < 0.05），说明抑制FAP可以显著降低CAFs的恶性表征。激光共聚焦结果显示外泌体能够被A549细胞所摄取。划痕实验与Transwell实验显示SP13786可抑制CAFs-exo对A549细胞迁移和侵袭的促进作用（*P* < 0.05）。与CAFs组比较，SP13786组A549细胞E-cadherin表达增多，N-cadherin与Slug表达降低（*P* < 0.05）；免疫荧光与Western blot显示SP13786组A549细胞的P-Stat3较CAFs组明显降低（*P* < 0.05），而总Stat3无显著差异。Stat3的特异性抑制剂WP1066明显抑制CAFs组A549细胞上皮间质转化（epithelial-mesenchymal transition, EMT），P-Stat3显著降低（*P* < 0.05），而加入WP1066后再加入SP13786-exo，P-Stat3未见进一步减低，EMT的抑制亦未见显著变化（*P* > 0.05）。

**结论:**

FAP的小分子特异性抑制剂SP13786通过影响CAFs外泌体间接抑制A549细胞的迁移、侵袭，其可能机制是抑制Stat3的磷酸化从而影响A549细胞的EMT。

## Abstract (en)

**Background and objective:**

Fibroblast activation protein (FAP) is one of the surface markers of cancer-associated fibroblasts (CAFs) and is closely related to the malignant characterization of CAFs. SP13786 is a specific micromolecule inhibitor of FAP and this study is to investigate the effects and mechanism of SP13786 on the migration and invasion of A549 cells through regulating exosomes of CAFs.

**Methods:**

CAFs and paracancerous fibroblasts (PTFs) were isolated and subcultured from freshly resected lung adenocarcinoma tissues and paracancerous normal tissues separately. MTT assay was used to detect the proliferation of CAFs incubated by different concentrations of SP13786; PTFs-exo, CAFs-exo and CAFs+SP13786-exo were extracted by polymer precipitation method. The A549 cells were divided into Ctrl group, PTFs group, CAFs group and SP13786 group and each group was incubated with DMEM, PTFs-exo, CAFs-exo and CAFs+SP13786-exo separately. Laser confocal microscope was used to observe the endocytoses of exosomes by A549 cells. The expression of alpha-smooth muscle actin (α-SMA) and FAP in PTFs and CAFs and the expression of E-cadherin, N-cadherin, Slug, Stat3 and P-Stat3 in A549 cells were detected by immunofluorescence, immunohistochemistry and Western blot. The migration and invasion ability of A549 cells were detected by cell scratch and transwell methods.

**Results:**

α-SMA and FAP were expressed much higher in CAFs than that in PTFs which indicate that CAFs and PTFs were successfully obtained from lung adenocarcinoma and paracancerous tissues (*P* < 0.05). MTT showed that the 50% inhibitory concentration (IC_50_) of SP13786 for CAFs was about 3.3 nmol/L. In addition, SP13786 can significantly decrease the expression of α-SMA and FAP in CAFs which means that targeted inhibition of FAP could reduce the malignant characteristics of CAFs (*P* < 0.05). Laser confocal microscope found that exosomes from CAFs could be taken up by A549 cells and scratch and transwell tests showed that the endocytosed CAFs-exo could promote the migration and invasion of A549 cells (*P* < 0.001), while FAP inhibitor SP13786 could inhibit the effects of CAFs-exo on A549 cells (*P* < 0.05). Furthermore, Immunofluorescence and Western blot showed that CAFs-exo could promote EMT by decreasing E-cadherin expression and increasing N-cadherin, Slug expression in A549 cells while FAP inhibitor SP13786 could significantly supress CAFs-exo-induced epithelial-mesenchymal transition (EMT) of A549 cells (*P* < 0.05). Moreover, the expression of P-Stat3 was obviously increased in A549 cells of CAFs group and significantly down-regulated in SP13786 group (*P* < 0.05) whereas there was no significant difference in total Stat3 between CAFs and SP13786 groups (*P* > 0.05). Finally, WP1066 (a specific inhibitor of Stat3) was used to comfirm whether SP13786 could influence EMT of A549 cells by inhibiting Stat3 phosphorylation via CAFs-Exo. The results showed that when the phosphorylation of Stat3 in CAFs group was inhibited by WP1066, SP13786 could not influence the P-Stat3 expression and EMT of A549 cells anymore (*P* > 0.05).

**Conclusion:**

As a specific micromolecule inhibitor of FAP, SP13786 indirectly inhibits the migration and invasion of A549 cells by affecting exosomes of CAFs. The possible mechanism is to inhibit the phosphorylation of Stat3 and thus affect the EMT of A549 cells.

肺癌是目前世界上最常见的恶性肿瘤之一^[1, 2]^。肺腺癌（lung adenocarcinoma, LUAD）是肺癌中最常见的病理类型^[3]^，侵袭转移是导致肺腺癌患者死亡的主要原因，如何抑制癌细胞的侵袭转移一直是肿瘤治疗的研究热点。

越来越多的证据表明肿瘤的发生发展与肿瘤微环境（tumor microenvironment, TME）关系密切。TME由肿瘤细胞、肿瘤相关成纤维细胞（cancer-associated fibroblasts, CAFs）、免疫细胞、间充质细胞以及间质和浸润在其中的生物分子等组成^[4, 5]^。CAFs是TME的重要组成成分，参与肿瘤细胞增殖、免疫逃逸、血管生成、淋巴转移和耐药等多个过程，已被认为是抗癌治疗的潜在靶点^[6, 7]^。研究^[8]^表明，α-平滑肌肌动蛋白（α-smooth muscle actin, α-SMA）、成纤维细胞活化蛋白（fibroblast activation protein, FAP）和成纤维细胞特异性蛋白（fibroblast specific protein, FSP）等分子在CAFs中表达较高，可以作为定义CAFs的标志物，Kalluri等^[9]^研究发现α-SMA和FAP在CAFs中更具特异性。

FAP是II型丝氨酸蛋白酶家族成员之一，具有二肽基肽酶及胶原酶活性，是CAFs表面的重要标志物，也是研究较多的CAFs治疗靶标。据报道FAP可以促进肿瘤的发生发展^[10, 11]^，提示抑制FAP可能有益于癌症治疗，然而靶向抑制CAFs中的FAP对肺腺癌迁移侵袭的作用及其机制尚不清楚。

外泌体是双层脂质构成的微囊（30 nm-150 nm），含有多种生物活性分子，包括DNA、microRNAs、蛋白质和脂质^[12, 13]^。外泌体是肿瘤细胞与微环境间信息交流的关键中介，在肿瘤发生、生长、血管生成、免疫逃逸、耐药性和迁移侵袭中发挥重要作用^[14-16]^。有学者^[17]^发现CAFs释放的外泌体可以促进肺腺癌细胞的迁移及侵袭，但特异性FAP抑制剂SP13786是否可以通过影响CAFs的外泌体从而抑制肺腺癌的迁移侵袭尚未见报道。

本研究发现CAFs外泌体可以上调A549细胞Stat3的磷酸化水平从而促进A549细胞的上皮间质转化（epithelial-mesenchymal transition, EMT）、迁移及侵袭，而FAP特异性抑制剂SP13786可以显著抑制CAFs外泌体诱导的Stat3-EMT，抑制A549细胞的迁移侵袭，提示SP13786可能是一个针对肺腺癌的潜在新型治疗药物。

## 1 材料与方法

### 1.1 材料与试剂

DMEM培养基购自美国Hyclone公司；SP13786[说明书标注半数抑制浓度（50% inhibitory concentration, IC_50_）为3.2 nmol/L]购自美国MCE公司；α-SMA一抗购自美国Sigma公司；FAP一抗购自英国Abcam公司；GAPDH、E-cadherin、N-cadherin、Slug、Stat3、P-Stat3一抗均购自美国CST公司；细胞上清外泌体提取试剂盒购自美国Invitrogen公司；基质胶购自美国BD公司；BCA蛋白定量试剂盒购自上海碧云天公司；DAPI购自Solarbio公司；Transwell小室购自美国Corning公司；SP9000免疫组化试剂盒购自北京中杉生物技术有限公司。

### 1.2 细胞培养

人肺腺癌细胞系A549购自中国科学院细胞库。培养基为含10%胎牛血清的DMEM培养基，于5% CO_2_细胞培养箱中培养，待细胞生长至对数期时进行传代。

原代肺腺癌CAFs及PTFs分别取自手术新鲜切除的肺腺癌组织和癌周正常组织。将新鲜的组织剪碎放在含0.12% I型胶原酶中，37 ℃消化30 min，离心1, 000 rpm、5 min，在5%CO_2_细胞培养箱中用含10%胎牛血清的DMEM培养基培养。通过形态观察及检测α-SMA和FAP判断是否成功获得CAFs与PTFs，将2代-5代细胞用于后续研究。

### 1.3 MTT实验

取对数生长期的CAFs细胞，以5×10^3^个/孔的细胞密度接种到96孔板中培养24 h，加入浓度为0 nmol/L、1.5 nmol/L、3 nmol/L、4.5 nmol/L、6 nmol/L和7.5 nmol/L的SP13786溶液，作用48 h后，每孔加20 μL MTT，继续培养4 h，加入150 μL DMSO，酶标仪检测570 nm波长处各孔的吸光度。重复3次，取平均值。

### 1.4 外泌体的分离和纯化

待PTFs、CAFs及CAFs+SP13786（3.3 nmol/L, 48 h）细胞密度生长至80%左右时，更换为含10%去外泌体胎牛血清的培养基，继续培养24 h，收集细胞培养液，2, 000 rpm离心30 min后取上清，并添加0.5倍体积的总外泌体分离试剂，4 ℃过夜。次日，4 ℃、10, 000 rpm离心1 h，弃上清，1×PBS重悬，即分别得到PTFs-exo、CAFs-exo以及CAFs+SP13786-exo。

### 1.5 透射电镜观察外泌体

分别将PTFs-exo、CAFs-exo以及CAFs+SP13786-exo与4%多聚甲醛混合，滴到Formvar碳涂层的电子显微镜格栅上，1%戊二醛固定10 min，2%乙酸铀酰溶液负染色。使用透射电镜在163 kV下获得图像。

### 1.6 荧光共聚焦显微镜观察A549细胞摄取外泌体

分别用PBS将PTFs-exo、CAFs-exo和CAFs+SP13786-exo稀释到1 mL，加入5 μL DID染料，37 ℃孵育30 min，1, 500 rpm离心5 min，弃上清用PBS重悬，分别加入到A549细胞中共培养0 h、24 h、48 h，荧光显微镜观察A549细胞摄取外泌体的情况。

### 1.7 A549细胞实验分组

Ctrl组；PTFs组：PTFs-exo与A549共孵育48 h；CAFs组：CAFs-exo与A549共孵育48 h；SP13786组：CAFs+SP13786-exo与A549共孵育48 h。

### 1.8 细胞免疫荧光实验

将CAFs、PTFs、CAFs+SP13786及不同处理组的A549细胞接种于共聚焦皿中，培养24 h，用4%多聚甲醛固定细胞15 min，山羊血清封闭1 h，分别加入α-SMA（1:400）、FAP（1:400）、Stat3（1:300）、P-Stat3（1:200）、N-cadherin（1:200）、E-cadherin（1:200）、Slug（1:200）抗体在4 ℃过夜。次日以荧光二抗（1:200）37 ℃孵育1.5 h，DAPI避光孵育8 min，封片，荧光共聚焦显微镜拍照。

### 1.9 细胞免疫组织化学实验

将CAFs、PTFs、CAFs+SP13786及不同处理组的A549细胞接种于24孔板中，培养24 h后用4%多聚甲醛固定15 min，3%H_2_O_2_ 15 min，山羊血清封闭20 min，分别加入下列一抗：α-SMA（1:400）、FAP（1:400）、Stat3（1:300）、P-Stat3（1:200）、N-cadherin（1:200）、E-cadherin（1:200），4 ℃过夜。次日，二抗37 ℃孵育30 min，DAB 8 min，苏木素10 min，拍照。

### 1.10 Western blot实验

将各组细胞提取蛋白，经SDS-PAGE凝胶分离后转移到PVDF膜上，5%脱脂奶粉封闭1.5 h，分别在α-SMA（1:2, 500）、FAP（1:5, 000）、Stat3（1:1, 000）、P-Stat3（1:1, 000）、N-cadherin（1:1, 000）、E-cadherin（1:1, 000）、Slug（1:1, 000）、Snail（1:1, 000）、β-actin（1:5, 000）和GAPDH（1:3, 000）中孵育，4 ℃过夜。次日用辣根过氧化物酶标记的二抗室温孵育1 h，ECL化学发光，凝胶成像系统曝光检测。

### 1.11 Transwell迁移及侵袭实验

将基质胶与无血清培养基以1:20稀释，包被及水化Transwell小室基底膜。将各组A549细胞用无血清培养基重悬，并调整细胞密度约为1×10^5^个/mL。在Transwell小室的上室每孔加入200 μL细胞悬液（约2×10^4^个细胞），下室加500 μL含10%FBS的DMEM培养基，37 ℃培养24 h，取出小室，4%多聚甲醛固定20 min，0.1%结晶紫染色20 min，拍照，计数细胞迁移数，并进行统计学分析。迁移试验不需要基质胶。

### 1.12 细胞划痕实验

取对数生长期的A549细胞接种于六孔板中，与各组外泌体共培养48 h，待细胞汇聚度达到80%，用灭菌蓝枪头进行划痕。于0 h、24 h分别用显微镜拍照，计算划痕愈合率。Image J软件计算细胞迁移率（%）= [（0-24）h的面积/0 h的起始面积]×100%。

### 1.13 统计学分析

采用SPSS 21.0软件及Image J软件进行实验数据的分析。计量资料用均数±标准差（Mean±SD）表示，组间比较采用单因素方差分析（*ANOVA*），利用GraphPad Prism 5进行图表绘制，*P* < 0.05为差异有统计学意义。

## 2 结果

### 2.1 CAFs和PTFs的分离与鉴定

CAFs和PTFs均为长梭形，且CAFs比PTFs形态更加细长（图 1A）。免疫荧光与免疫组化结果均显示α-SMA和FAP在CAFs中高表达而在PTFs中低表达（图 1B，图 1C）。表明通过原代分离培养成功获得了人肺腺癌CAFs和PTFs。

**图 1:**
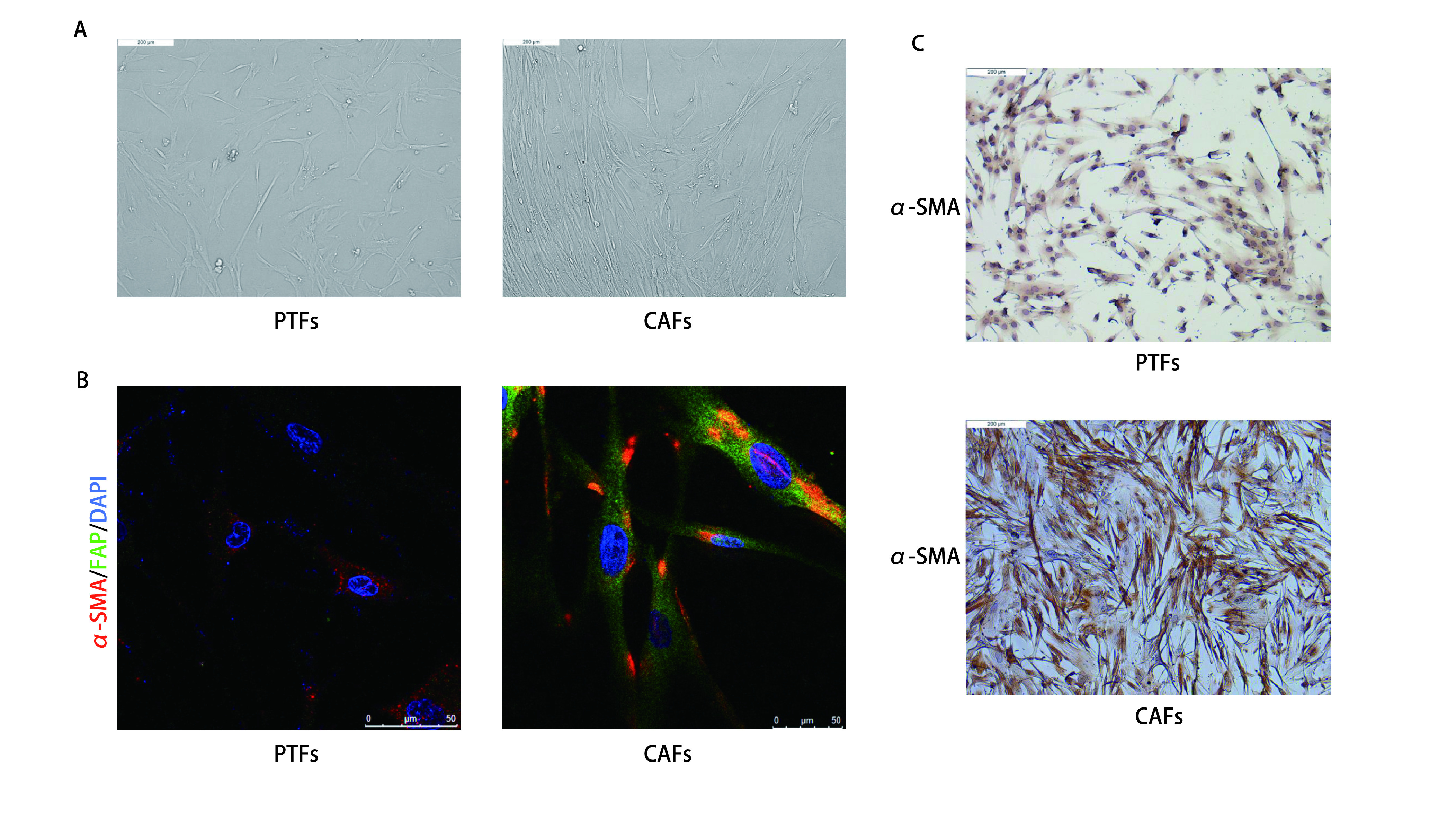
原代分离的CAFs和PTFs形态及表型鉴定。A：显微镜下观察CAFs及PTFs的细胞形态（×100）；B：免疫荧光检测α-SMA及FAP在CAFs和PTFs中的表达；C：免疫组化检测α-SMA在CAFs和PTFs中的表达量（×100）。 Morphology and phenotypic identification of primary isolated CAFs and PTFs. A: Morphological features of primary cultured PTFs and CAFs (×100). B: The expression levels of α-SMA and FAP in CAFs and PTFs were analyzed by immunofluorescence technique. The slide was stained with 4', 6-diamidino-2-phenylindole (DAPI, blue), FAP antibody (green) and α-SMA antibody (red); C: The expression levels of FAP in CAFs and PTFs were analyzed by immunohistochemistry (×100). CAFs: cancer-associated fibroblasts; PTFs: peri-tumer fibroblasts; α-SMA: alpha-smooth muscle actin; FAP: fibroblast activation protein.

### 2.2 FAP抑制剂SP13786显著抑制CAFs中FAP的表达

用不同浓度的SP13786孵育CAFs 48 h并进行MTT实验以检测CAFs增殖率。如图 2A所示，SP13786显著降低CAFs的增殖，其IC_50_值约为3.3 nmol/L。Western blot结果显示与CAFs组相比，CAFs+SP13786组的FAP与α-SMA的表达水平明显降低（*P* < 0.05，图 2B）。进一步通过免疫荧光与免疫组化实验得到相同结果，如图 2C、图 2D。表明SP13786（3.3 nmol/L）孵育CAFs 48 h后CAFs中FAP与α-SMA的表达显著降低，CAFs向正常成纤维细胞转化，从而选用3.3 nmol/L的SP13786用于后续实验。

**图 2:**
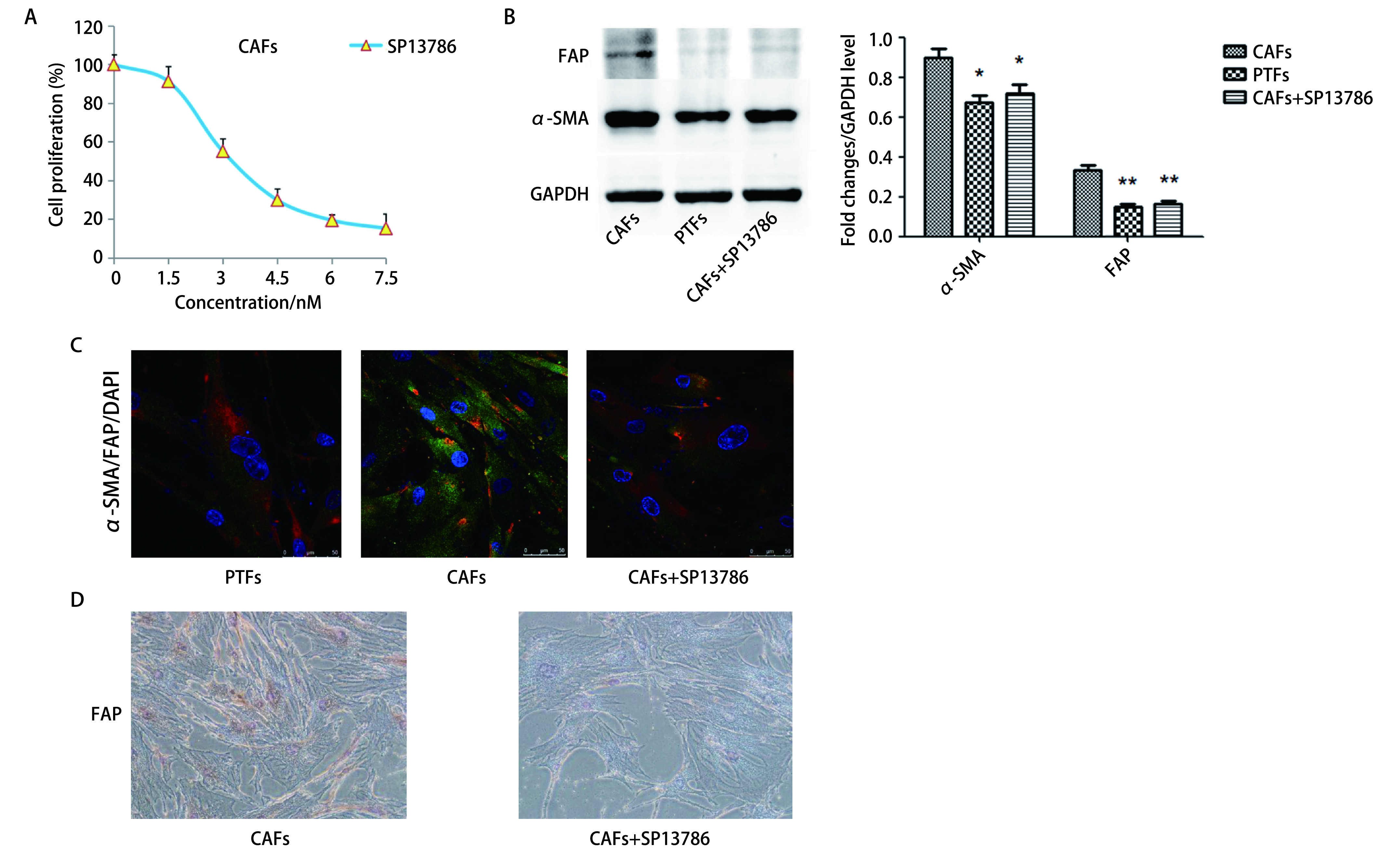
SP13786对CAFs的增殖及FAP、α-SMA表达的影响。A：MTT检测CAFs增殖率；B、C：Western blot和免疫荧光检测α-SMA及FAP的表达；D：免疫组化检测FAP的表达（×200）。*P < 0.05；**P < 0.01。 Effects of SP13786 on the proliferation of CAFs and the expression of FAP and α-SMA. A: MTT was used to detect the proliferation rate of CAFs; B, C: The expression of α-SMA and FAP was detected by Western blot (B) and immunofluorescence (C); D: Expression of FAP detected by immunohistochemistry (×200). *P < 0.05; **P < 0.01.

### 2.3 外泌体鉴定与细胞摄取

分别提取PTFs-exo、CAFs-exo以及CAFs+SP13786-exo。透射电子显微镜发现样品均呈椭圆形或球形囊泡，其粒径大小在40 nm-150 nm之间（图 3A）。将各组外泌体与A549细胞共孵育0 h、24 h和48 h，荧光共聚焦结果显示外泌体能够被A549细胞所摄取且摄取量随时间延长而增多（图 3B）。

**图 3:**
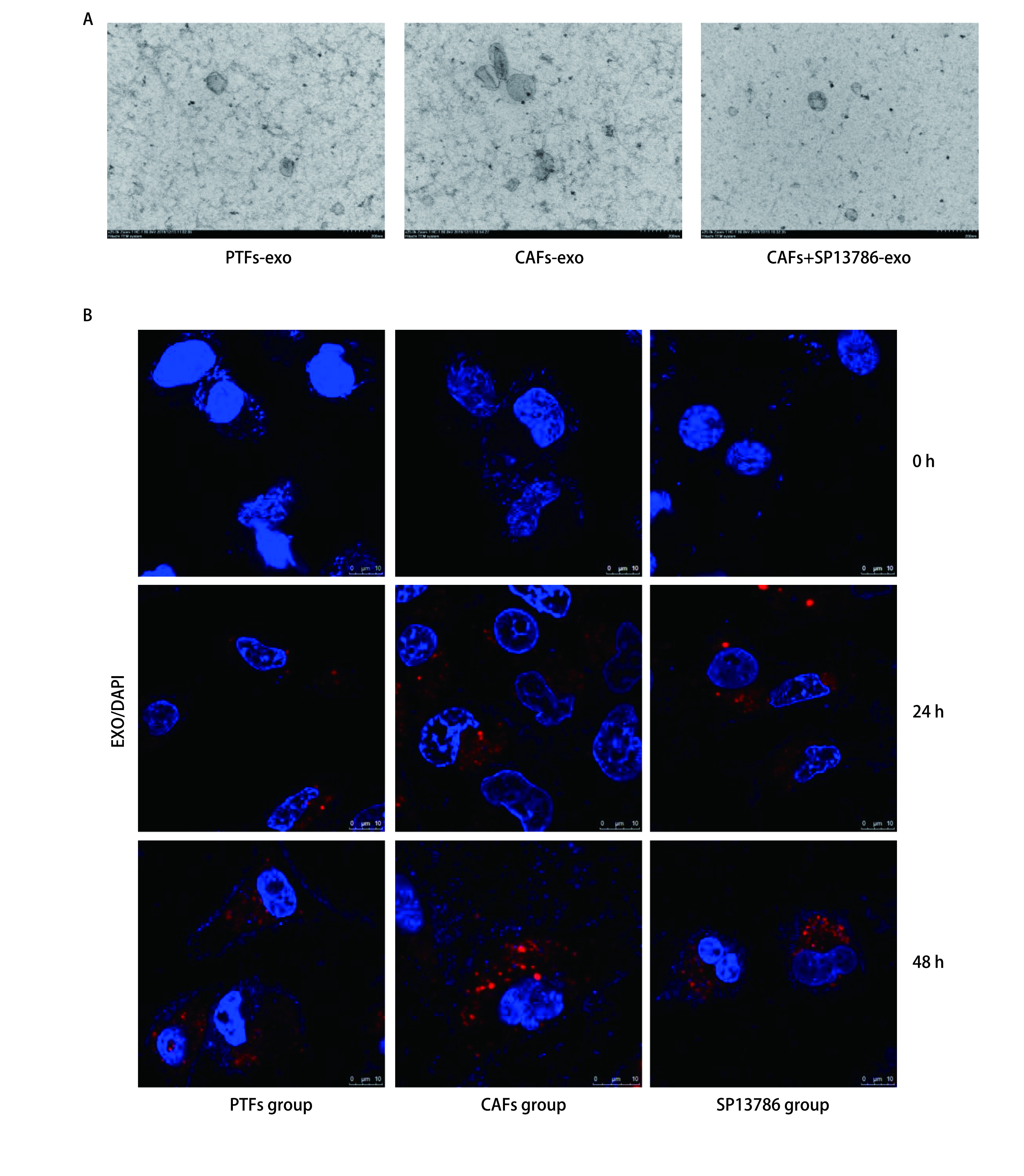
外泌体的鉴定与细胞摄取。A：PTFs-exo、CAFs-exo和CAFs+SP13786-exo的透射电镜照片；B：PTFs-exo、CAFs-exo和CAFs+SP13786-exo（红色）进入A549细胞。 Identification of exosomes and intercellular metastasis. A: Transmission electron micrograph of PTFs-exo, CAFs-exo and CAFs+SP13786-exo; B: The PTFs-exo, CAFs-exo and CAFs+SP13786-exo (red) were absorbed by A549 cells.

### 2.4 SP13786通过CAFs外泌体影响A549细胞形态、迁移及侵袭

与Ctrl组比较，CAFs组的A549细胞呈纺锤形变化趋势（图 4A）。Transwell迁移及侵袭实验结果均显示CAFs组A549细胞的穿膜数目较Ctrl组显著增多（*P* < 0.001），而SP13786组较CAFs组穿膜细胞数显著减少（*P* < 0.05），与PTFs组比较差异无统计学意义（*P* > 0.05，图 4B，图 4C）。划痕实验结果显示CAFs组A549细胞划痕愈合能力较Ctrl组显著增强（*P* < 0.001），而SP13786组细胞的划痕愈合能力较CAFs组显著下降（*P* < 0.05），与PTFs组比较差异无统计学意义（*P* > 0.05，图 4D）。上述结果表明SP13786可通过CAFs外泌体间接影响A549细胞的形态与迁移侵袭。

**图 4:**
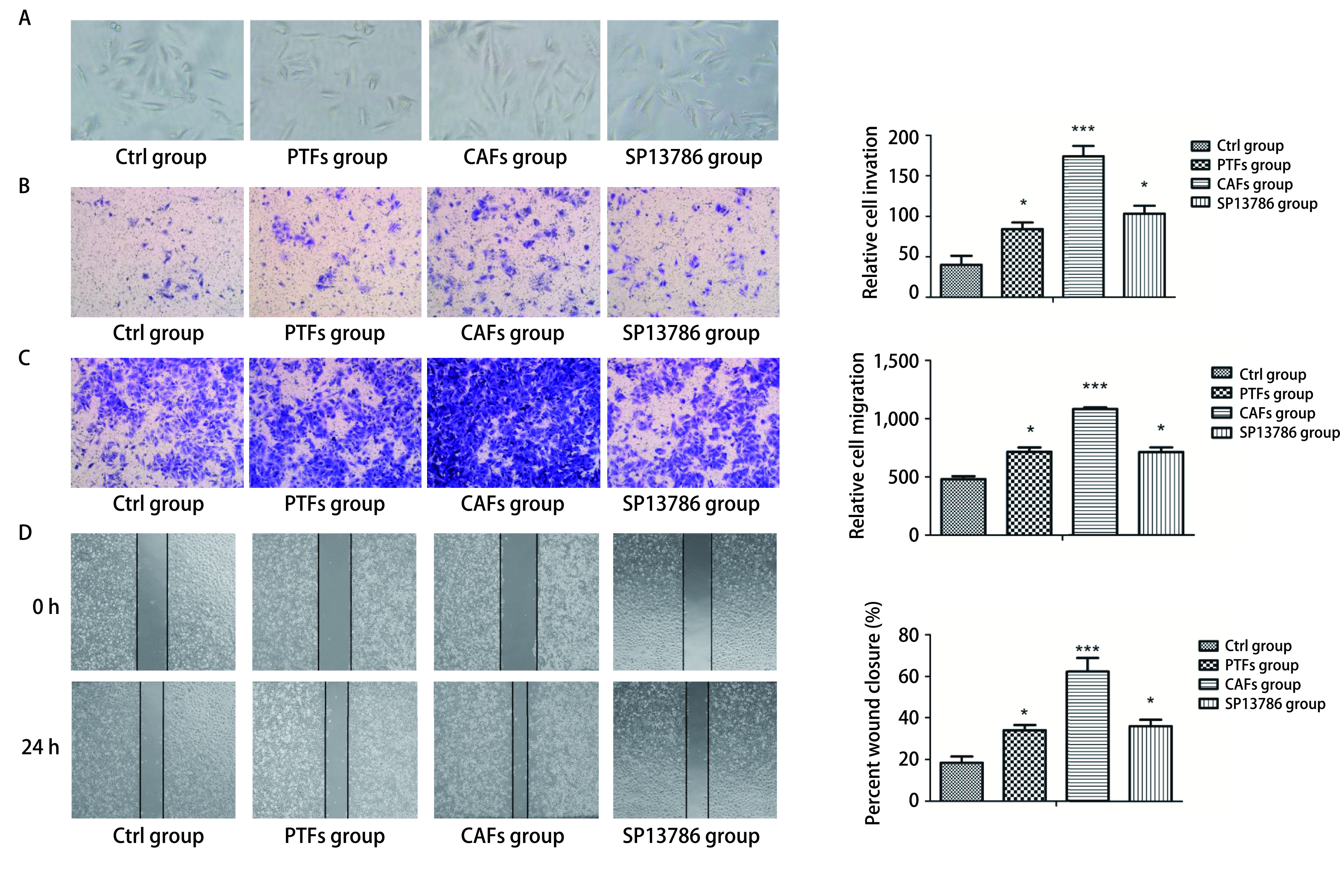
SP13786通过CAFs-exo对A549细胞形态、迁移及侵袭的影响。A：PTFs-exo、CAFs-exo和CAFs+SP13786-exo对A549细胞形态的影响（×100）；B：Transwell检测24 h后PTFs-exo、CAFs-exo和CAFs+SP13786-exo对A549细胞侵袭能力的影响（×200）；C、D：Transwell迁移实验（C, ×200）和划痕实验（D, ×40）检测24 h后PTFs-exo、CAFs-exo和CAFs+SP13786-exo对A549细胞迁移能力的影响。*P < 0.05；***P < 0.001。 Effects of SP13786 on the morphology, invasion and migration of A549 cells through CAFs-exo. A: The effect of PTFs-exo, CAFs-exo and CAFs+SP13786-exo on the morphology of A549 cells (×100); B: Transwell assays were performed to evaluate cell invasive ability after 24h (×200); C, D: Transwell assays (C, ×200) and wound healing assays (D, ×40) were performed to evaluate cell migration ability after 24 h. *P < 0.05; ***P < 0.001.

### 2.5 SP13786通过抑制Stat3的磷酸化抑制EMT

为进一步明确SP13786的作用机制，我们观察了CAFs-exo对A549细胞EMT的影响。免疫荧光检测发现，与Ctrl组相比，CAFs组A549细胞的E-cadherin表达降低，N-cadherin表达增加（*P* < 0.01）；与CAFs组比较，SP13786组A549细胞的E-cadherin表达增加，N-cadherin表达减少（*P* < 0.05），而与PTFs组比较则无显著差异（*P* > 0.05，图 5A）。Slug与Stat3是调控EMT的重要转录因子，CAFs组Slug与P-Stat3表达较Ctrl组均显著增多（*P* < 0.01），SP13786组Slug与P-Stat3表达均较CAFs组减少（*P* < 0.05），与Ctrl组相比均未见显著差异（*P* > 0.05，图 5A）。同时Western blot（图 5B）与免疫组化（图 5C）均显示出与免疫荧光相同的结果。表明CAFs-exo可以促进A549细胞EMT，SP13786可以抑制CAFs-exo对A549细胞EMT的促进作用。为了确定SP13786是否是通过抑制Stat3磷酸化而抑制了A549细胞的EMT，我们进一步使用Stat3抑制剂WP1066进行验证。结果显示CAFs组加入WP1066后，A549细胞E-cadherin表达增高，N-cadherin表达降低，P-Stat3显著减少（*P* < 0.05）；在CAFs组中同时加入WP1066与SP13786后，E-cadherin、N-cadherin及P-Stat3表达与只加入WP1066无显著差异（*P* > 0.05，图 5D）。提示CAFs-exo可通过诱导A549中Stat3的磷酸化促进EMT的转化，增强A549细胞的迁移侵袭，而SP13786可通过影响CAFs-exo间接抑制A549细胞中Stat3磷酸化从而抑制EMT。

**图 5:**
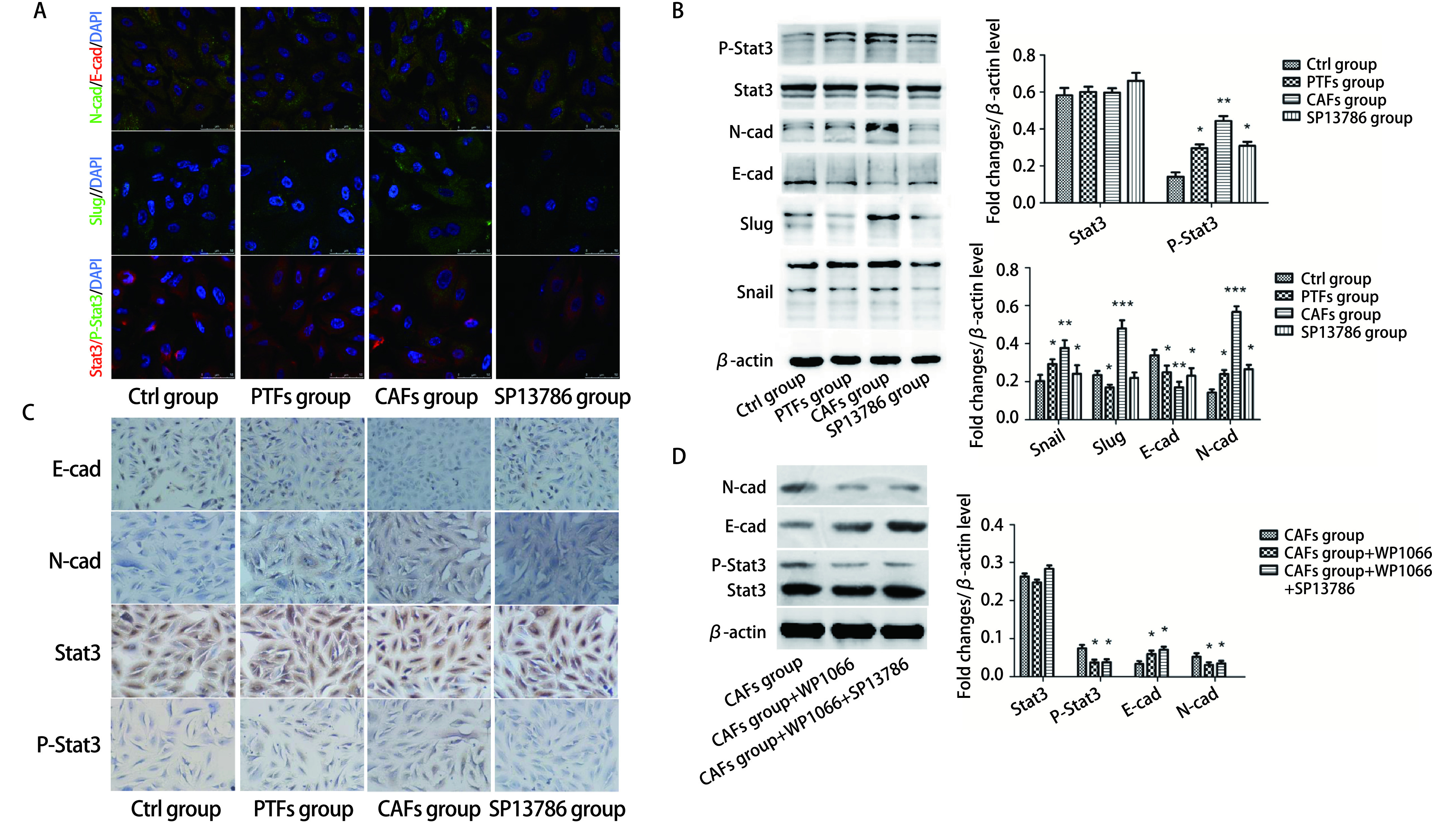
SP13786通过CAFs-exo对A549细胞EMT及Stat3磷酸化的影响。A-C：免疫荧光、Western blot和免疫组化检测E-cadherin、N-cadherin、Slug、Stat3及P-Stat3的表达量；D：Western blot检测E-cadherin、N-cadherin、Stat3及P-Stat3的表达量。*P < 0.05；**P < 0.01；***P < 0.001。 Effects of SP13786 on EMT and Stat3 phosphorylation in A549 cells via CAFs-exo. A-C: The expression levels of E-cadherin, N-cadherin, Slug, Stat3 and P-Stat3 were detected by immunofluorescence (A), Western blot (B) and immunohistochemistry (C, ×200); D: Western blot to detect the expression of E-cadherin, N-cadherin, Stat3 and P-Stat3. *P < 0.05; **P < 0.01; ***P < 0.001. E-cad: E-cadherin; N-cad: B-cadherin.

## 3 讨论

肺腺癌是肺癌中最常见的病理类型^[18, 19]^，侵袭转移是导致患者死亡的主要原因。研究表明，肿瘤的侵袭转移不只与肿瘤细胞的特性有关，还受肿瘤微环境的影响。CAFs是一种活化的成纤维细胞，是肿瘤微环境的主要细胞成分，通过多种通信方式促进肿瘤的发展和迁移^[9, 20]^。为了研究CAFs在肺腺癌迁移侵袭中的作用与机制，本研究分别从新鲜切除的肺腺癌组织和癌旁正常组织中分离提取并培养了CAFs和PTFs，免疫荧光和免疫组化结果显示CAFs中α-SMA和FAP的表达水平显著高于PTFs，且CAFs比PTFs形态更加细长，这一结果与文献^[20, 21]^报道结果一致。

FAP是丝氨酸蛋白酶家族的一种II型跨膜细胞表面蛋白。FAP不仅在间质成纤维细胞表达，也在各种上皮来源的癌细胞中存在，对多种恶性肿瘤的发生发展产生影响^[22-24]^，是潜在的肿瘤早期诊断标志物与治疗靶点^[25, 26]^。近年来，靶向FAP在肿瘤治疗中的应用越来越受到关注。比如抗FAP单克隆抗体FAP5-DM1与美登木素生物碱联用，可以抑制癌症的进展^[27]^；靶向FAP的免疫毒素FAP-PE38在乳腺癌中可以特异性消除CAF中的FAP，并显示出抑制肿瘤发展的作用^[28]^。有研究^[29-31]^报道FAP抑制剂如Talabostat及Sibrotuzumab等能通过抑制FAP对肿瘤产生抑制作用，但因未能通过临床二期试验，仍需继续探索以寻找更有效的FAP抑制剂。新型小分子FAP抑制剂SP13786具有抗肿瘤作用^[32]^，但其对肺腺癌的影响尚未见报道。本研究观察到3.3 nmol/L浓度的SP13786通过特异性抑制FAP使CAFs的标志物FAP和α-SMA表达显著降低，使CAFs向正常成纤维细胞转化，因此我们推测SP13786可以通过影响CAFs的功能，改变肿瘤微环境从而起到抑制肿瘤发生发展的作用。

外泌体是细胞间沟通的重要媒介，CAFs可通过外泌体影响肿瘤细胞的功能。为明确SP13786对CAFs外泌体功能的影响，本研究分别提取了PTFs-exo、CAFs-exo和CAFs+SP13786-exo。结果表明，外泌体被A549细胞摄取后，CAFs组的A549细胞侵袭迁移能力较PTFs组显著增强，而SP13786组的迁移及侵袭能力较CAFs组明显减弱，提示SP13786靶向抑制CAFs中的FAP后减弱了CAFs-exo的恶性表征，从而降低了CAFs外泌体对A549细胞迁移侵袭的促进作用。EMT是上皮细胞失去连接和极性并转变为间充质表型的基本生物学过程^[33]^，在肿瘤的迁移中发挥着重要作用。Shintani等^[20, 34]^的研究发现CAFs可以诱导肺癌细胞发生EMT，本研究也发现CAFs-exo可以促进A549细胞EMT，应用SP13786抑制FAP后，CAFs-exo促进A549细胞EMT的能力显著减弱，SP13786组A549细胞与CAFs组比较，E-cadherin增加，N-cadherin和Slug降低，说明SP13786能够通过影响CAFs-exo间接影响A549细胞的EMT与迁移侵袭。

Stat3通过促进肿瘤细胞EMT进而增加肿瘤细胞的迁移、侵袭能力^[35, 36]^。本研究结果显示CAFs-exo使CAFs组A549细胞的P-Stat3显著增加，而SP13786组A549细胞中P-Stat3与CAFs组比较显著减少，提示SP13786可以通过影响CAFs-exo间接抑制A549细胞中Stat3的磷酸化而逆转EMT，抑制A549细胞的迁移和侵袭。有趣的是，应用Stat3的特异性抑制剂WP1066抑制CAFs组A549细胞Stat3活化后，SP13786不能对Stat3的磷酸化以及A549细胞的EMT起到进一步的抑制作用，进一步证明SP13786可能是通过影响CAFs-exo间接抑制A549细胞Stat3的活化从而抑制A549细胞的EMT及迁移侵袭。

综上所述，本研究通过细胞实验证明FAP的特异性小分子抑制剂SP13786能够通过影响CAFs的外泌体间接抑制A549细胞Stat3的磷酸化，进而抑制A549细胞的EMT、迁移和侵袭，提示FAP可能成为肺腺癌治疗的新靶点，SP13786有望成为肺腺癌治疗的潜在新型药物。但本研究尚未对SP13786作用前后CAFs外泌体内成分的变化做出阐明，也未扩展到动物实验进行验证，具有一定的局限性，有待进一步深入研究。
